# *Sebastidae*-specific food allergy causing anaphylaxis: a pediatric case report and fish allergen analysis

**DOI:** 10.1186/s12887-026-06987-0

**Published:** 2026-05-14

**Authors:** Mitsuru Tsuge, Kazuhiro Uda, Masanori Ikeda, Hirokazu Tsukahara

**Affiliations:** 1 Department of Pediatric Acute Diseases, Okayama University Graduate School of Medicine, Dentistry, and Pharmaceutical Sciences, 2-5-1 Shikata-cho, Kita-ku, Okayama, 700-8558 Japan; 2 Department of Pediatrics, Okayama University Graduate School of Medicine, Dentistry, and Pharmaceutical Sciences, Okayama, Japan; 3 Department of Pediatrics, Okayama University Hospital, Okayama, Japan

**Keywords:** Food allergy, Fish allergy, *Sebastidae*, Basophil activation test

## Abstract

**Background:**

Fish allergy affects approximately 0–0.3% of the population. Parvalbumin is the major allergen in fish allergy, and patients with fish allergy sensitized to parvalbumin demonstrate allergic reactions to various fish species. However, a subset of patients exhibits allergic responses to only specific fish species. Fish of the *Sebastidae* family, widely consumed in Asian, North American, and European countries, has rarely been reported as an allergenic trigger.

**Case presentation:**

A 14-year-old Japanese boy visited our clinic for evaluation of fish allergy after experiencing allergic reactions to *Sebastes alutus* (Alaskan rockfish). He developed respiratory symptoms, eyelid edema, and skin erythema after consuming a meal containing *Sebastes alutus*, suggesting anaphylaxis; however, he was able to consume many other fish species without any issues. Skin prick tests and basophil activation tests using fish species from the *Sebastidae* family showed positive reactions, whereas other fish species that the patient could tolerate showed negative reactions. Immunoblot analysis performed to identify the causative allergen revealed two IgE-binding bands (17 and 110 kDa) in *Sebastidae*, distinct from parvalbumin. Mass spectrometry identified these proteins as alpha-actinin-3b, ATPase sarcoplasmic/endoplasmic reticulum Ca2 + transporting 1, and several myosin-related proteins.

**Conclusions:**

This case highlights the need for clinicians to recognize fish allergy specific to the *Sebastidae* family. Identifying the causative non-parvalbumin allergens would improve understanding of fish allergy diversity and individualized dietary management for patients. Additional cases and detailed analyses are necessary to identify the specific allergens involved in *Sebastidae* allergy.

## Background

Fish is recognized as one of the major allergenic foods worldwide; however, the overall prevalence of fish allergy is relatively low, estimated to be approximately 0–0.3% in the general population based on oral food challenge–confirmed cases [1, 2]. The prevalence of fish allergy varies by region. In Japan, the overall prevalence of food allergy is estimated to be 1–2%, but data on fish allergy confirmed by oral food challenge are limited, and the exact prevalence remains unclear. Parvalbumin has been identified as the major allergen implicated in fish allergy. The majority of patients with fish allergy are sensitized to parvalbumin, and demonstrate allergic reactions to a broad spectrum of fish species. However, a subset of patients exhibits allergic responses to specific fish species while tolerating others [3]. In such cases, sensitization may be attributed to species-specific allergens unique to those particular fish.

The family *Sebastidae* comprises a large number of species, nearly all of which are consumed as food, making it an important global seafood resource. *Sebastidae* are prized for their mild flavor and ease of preparation, and are a staple in many traditional Asian, North American, and European cuisines. In Japan, they are commonly used in meals for infants and young children, including nursery and preschool meals; the species *Sebastes alutus* (Alaskan rockfish) and *Sebastes marinus* (Atlantic redfish), which are native to the Gulf of Alaska and North Atlantic, are most commonly prepared. Species of the *Sebastidae* family are widely referred to by the common name “rockfish”; however, it is also used to describe species belonging to several other families.

Herein, we present the case of a Japanese boy who developed anaphylaxis after ingesting fish species belonging to the *Sebastidae* family but was able to consume many other fish species without any issues. There have been few case reports of *Sebastidae* allergy, and this report includes an analysis of the *Sebastes*-specific allergens identified in this patient. This case report describes sensitization and the investigation of causative allergens in fish of the *Sebastidae* family, including species of the genus *Sebastes*.

## Case presentation

A 14-year-old boy presented to our outpatient department for investigation of fish allergy after experiencing allergic reactions following *Sebastes alutus* consumption. At the age of 12, approximately 30 min after ingesting a meal containing Japanese-style fried *Sebastes alutus* with a thickened sauce and pumpkin salad, he developed generalized skin erythema, cough, dyspnea, sore throat, and eyelid edema without any gastrointestinal symptoms. Upon admission, his vital signs revealed tachycardia (130 bpm), normal oxygenation (96%), normal blood pressure (102/56 mmHg), and an unaffected level of consciousness. The case was consistent with anaphylaxis; however, the attending physician did not diagnose the patient with anaphylaxis at that time, and intramuscular adrenaline was not administered. All symptoms resolved within one hour following antihistamine administration alone.

The meal he consumed included *Sebastes alutus*, carrots, bean sprouts, pumpkin, snow peas, shiitake mushrooms, soy sauce, sesame oil, bonito, kelp, potato starch, and rice. Of these components, *Sebastes alutus* was the only item he had never consumed previously, whereas all other foods were generally well tolerated. Based on the medical interview with the patient and his guardian, he had never consumed any fish from the *Sebastidae* family including *Sebastes alutus* throughout the 14 years prior to this episode. The patient was able to consume various other fish, including tuna, cod, sardines, horse mackerel, yellowtail, and salmon, without any adverse reactions. The patient and family requested a comprehensive evaluation to determine to which species of *Sebastidae* he was allergic.

The patient’s medical history included food allergies (chicken eggs, salmon roe, and shrimp) and allergic rhinitis; however, he had no history of atopic dermatitis, regular contact with fish, or stings by a fish fins. Laboratory tests revealed an elevated serum total IgE level (258 IU/mL). Serum-specific IgE antibody tests for seven fish species (tuna, horse mackerel, sardine, mackerel, flounder, salmon, and cod) were negative using the ImmunoCAP assay (Thermo Fisher Scientific, Uppsala, Sweden). No available serum-specific IgE test for *Sebastidae* exists commercially in Japan.

The fish samples analyzed in this study were commercially available raw fillets purchased from a retail market by the patient’s guardians for the skin prick test, basophil activation test, serum IgE reactivity assay, and immunoblot analysis described below.

The skin prick test (SPT) was performed using raw fish species from various fish species belonging to the *Sebastidae* family, including *Sebastes alutus*, *Sebastes inermis*, *Sebastes ventricosus*, *Sebastes oblongus*, *Sebastes pachycephalus*, *Sebastiscus marmoratus* (marbled rockfish), *Sebastiscus albofasciatus*, *Sebastolobus macrochir* (broadbanded thornyhead), as well as *Scorpaenopsis cirrhosa* (weedy scorpionfish). SPT responses were assessed at 15 min after application, and the wheal diameter was measured. All tested species were positive with wheal diameters of 10 × 11, 8 × 10, 14 × 10, 8 × 11, 11 × 14, 16 × 10, 6 × 10, 8 × 10, and 8 × 8 mm, respectively. In contrast, SPT reactions to *Thunnus albacares* (yellowfin tuna), *Gadus macrocephalus* (Pacific cod), and *Etrumeus teres* (round herring) were negative (0 × 0, 1 × 1, and 0 × 0 mm, respectively). The positive and negative controls, histamine hydrochloride (10 mg/mL) and saline, produced wheal diameters of 5 × 5 mm and 0 × 0 mm, respectively.

**Fig. 1:**
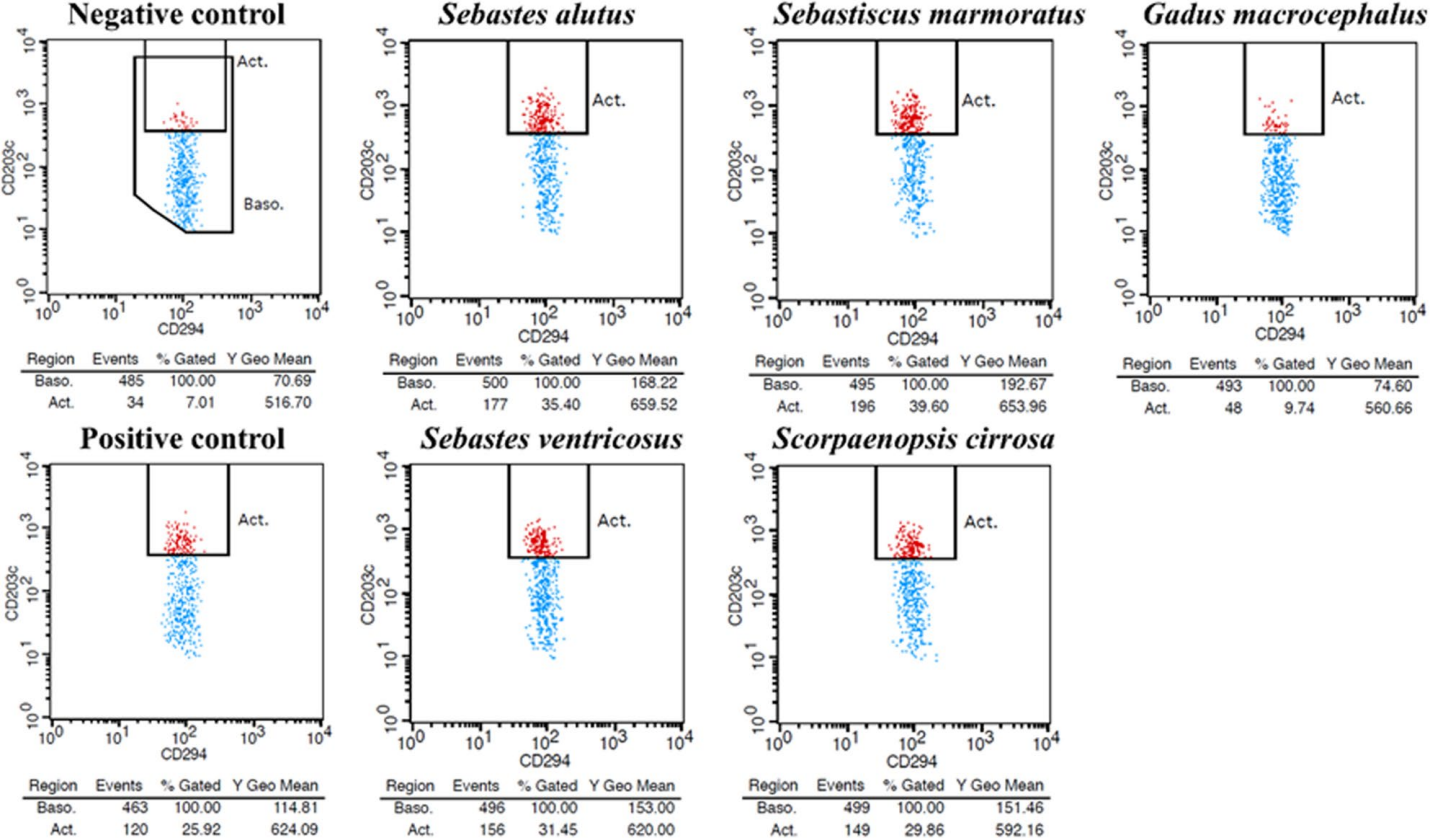
Whole-blood basophil activation test stimulated with fish extracts. Four fish species (*Sebastes alutus*, *Sebastes ventricosus*, *Sebastiscus marmoratus*, and *Scorpaenopsis cirrhosa*) were used for stimulation, along with *Gadus macrocephalus* as a negative control fish. The patient’s whole blood was incubated with each fish extract, and CD203c-expressing basophils were detected using fluorescence-activated cell sorting. The positive control was treated with an anti-immunoglobulin E antibody

Basophil activation test (BAT) was performed at an external facility (Bio Medical Laboratories, Saitama, Japan) to evaluate IgE-mediated allergic reactions to *Sebastidae*. CD203c expression in the activated basophils was quantified by culturing whole blood with *Sebastidae* antigens. Four fish species (*Sebastes alutus*, *Sebastes ventricosus*, *Sebastiscus marmoratus*, and *Scorpaenopsis cirrhosa*) were used for stimulation, with *Gadus macrocephalus* as a negative control fish. Each raw fish sample was subsequently homogenized in distilled water using an ultrasonic homogenizer, while the patient’s peripheral whole blood was incubated with the extracted samples. CD203c-expressing basophils were detected by fluorescence-activated cell sorting. BAT results showed 35.4% CD203c-positive cells with *Sebastes alutus*, 31.5% with *Sebastes ventricosus*, 39.6% with *Sebastiscus marmoratus*, and 29.9% with *Scorpaenopsis cirrhosa* at a concentration of 100 µg/mL (Fig. 1). In contrast, *Gadus macrocephalus* elicited 9.7% CD203c-positive cells. For positive (anti-immunoglobulin E) and negative controls, BAT revealed 25.9% and 7.0% CD203c-positive cells, respectively.

**Fig. 2:**
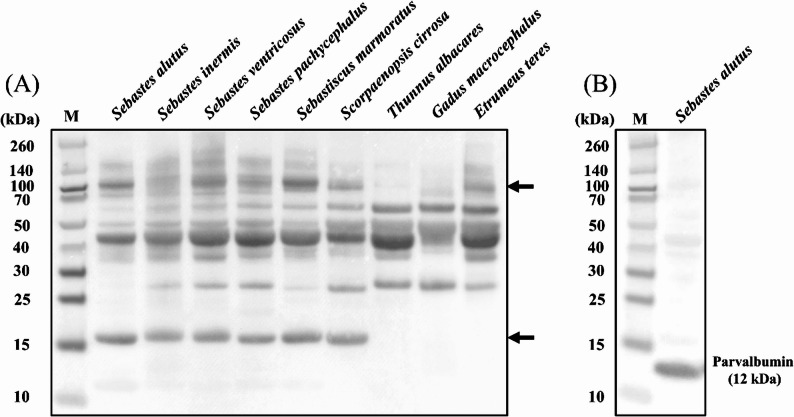
**A** Serum IgE reactivity in immunoblot analysis against fish protein extracts. Extracted proteins from each fish are separated by SDS-PAGE, transferred to a PVDF membrane, and probed with 10% of the patient’s serum. **B** Immunoblotting analysis against parvalbumin in extracts of *Sebastes alutus*. Lane (M) represents the molecular weight marker. SDS-PAGE, sodium dodecyl sulfate–polyacrylamide gel electrophoresis; PVDF, polyvinylidene difluoride; IgE, immunoglobulin E

To identify the causative allergic components in *Sebastidae*, serum IgE reactivity to proteins extracted from each fish was analyzed using immunoblot assay. Eight raw fish species (*Sebastes alutus*, *Sebastes inermis*, *Sebastes ventricosus*, *Sebastes pachycephalus*, *Sebastiscus marmoratus*, *Sebastiscus albofasciatus*, *Sebastolobus macrochir*, and *Scorpaenopsis cirrhosa*) were prepared for analysis. And three types of raw fish (*Thunnus albacares*,* Gadus macrocephalus*, and *Etrumeus teres*) that the patient normally consumed without allergic symptoms were also prepared as controls. Each fish was homogenized in a protein extraction solution (EzRIPA Lysis kit, ATTO Corporation, Tokyo, Japan) containing protease inhibitors at 4 °C. Equal amounts (30 µg) of extracted proteins were separated by sodium dodecyl sulfate–polyacrylamide gel electrophoresis under denaturing conditions and blotted onto polyvinylidene difluoride membranes. After blocking, the membranes were incubated overnight with 10% patient serum, as previously reported [3, 4]. The membranes were then treated with a mouse anti-human IgE Fc receptor monoclonal antibody conjugated with horseradish peroxidase (1:5,000 dilution, ab99806; Abcam, Cambridge, UK) and developed using the SuperSignal™ West Pico PLUS chemiluminescent substrate system (Thermo Fisher Scientific, Waltham, MA, USA). Immunoblot analysis revealed that the IgE antibodies in the patient’s serum reacted with two protein bands (approximately 17 and 110 kDa) of *Sebastidae* proteins, but not with the proteins of the negative control fish (Fig. 2A). IgE-reactive bands of various molecular weights were detected in all fish species, including non-causative ones; therefore, our analysis focused on the bands that appeared specifically in *Sebastidae* species. To compare the molecular weight of parvalbumin (12 kDa) from *Sebastes alutus*, immunoblotting was performed using a mouse anti-parvalbumin monoclonal antibody (1:2000 dilution; P3088, Sigma-Aldrich, St. Louis, MO, USA) (Fig. 2B). The molecular weights of these two bands differed from that of parvalbumin. A faint band of approximately 110 kDa was detected in the protein extracted from *Etrumeus teres*. A specific IgE test against Gad c 1 (cod parvalbumin, *Gadus callarias*) in the patient’s serum, measured using the ImmunoCAP assay, was negative.

Mass spectrometry was performed to investigate the protein bands of approximately 17 and 110 kDa. Protein bands excised from the gel were digested with trypsin and analyzed using nano-LC-MS/MS following a standard protocol (Japan Proteomics Co., Ltd., Sendai, Japan). Peptide identification was performed using the MASCOT Server (Matrix Science, London, UK). The MS/MS spectra were searched against the protein database of the *Sebastes* species, using sequence similarity-based analysis. As the available database consisted mainly of predicted protein sequences with limited annotation, additional reference to homologous protein sequences from *Danio rerio* (zebrafish) was used to support protein identification. For the 110 kDa band, alpha-actinin-3b and ATPase sarcoplasmic/endoplasmic reticulum Ca2 + transporting 1-like isoform X1 were identified. For the 17 kDa band, nucleoside diphosphate kinase B-like; fast skeletal muscle myosin light polypeptide 3, and myosin light chain, phosphorylatable, fast skeletal muscle a were identified (Table 1).

**Table 1:** Proteins identified by mass spectrometry from the immunoreactive gel bands

Excised band 1 (110 kDa)	Protein [Organism], Accession number	Molecular weight (Da)	Score*	Coverage (%)
1	alpha-actinin-3b [Sebastes umbrosus], XP_037615293.1	103,609	259	14
2	ATPase sarcoplasmic/endoplasmic reticulum Ca2 + transporting 1, like isoform X1 [Sebastes umbrosus], XP_037610831.1	108,827	97	6
Excised band 2 (17 kDa)	Protein [Organism], Accession number	Molecular weight (Da)	Score*	Coverage (%)
1	nucleoside diphosphate kinase B-like [Sebastes umbrosus], XP_037618593.1	16,802	310	75
2	myosin, light polypeptide 3, skeletal muscle [Sebastes umbrosus], XP_037618270.1	19,594	94	19
3	myosin light chain, phosphorylatable, fast skeletal muscle a [Sebastes umbrosus], XP_037647912.1	19,029	63	15

*Score indicates the MASCOT ion score reflecting the confidence of peptide identification

After eliminating all species of the *Sebastidae* family from the diet, with the informed consent of the patient and his parents, no allergic reactions occurred during the six-month follow-up period. Other fish species could still be consumed freely without allergic symptoms.

### Discussion and conclusions

Fish allergy caused by *Sebastes* species is globally rare, including in Japan. One reported case involved a one-year-old Japanese boy with a preexisting food allergy to *Trachurus japonicus* (Japanese jack mackerel) who developed allergic symptoms after ingesting *Sebastes matsubarae* [4]. Similarly, our patient was able to consume various other fish species, including tuna, salmon, cod, sardine, *Scomber japonicus* (Pacific chub mackerel), and *Seriola quinqueradiata* (Japanese amberjack), without any issues. However, detailed information was not provided regarding the presence of allergic reactions to other species of the *Sebastidae* family.

The *Sebastidae* family belongs to the suborder *Scorpaenoidei*, and includes several genera: *Sebastes*, *Sebastiscus*, *Sebastolobus*, *Hozukius*, and *Helicolenus*. *Scorpaenopsis cirrhosa* belongs to the family *Scorpaenidae*, which is phylogenetically related to the *Sebastidae* family. Compared with other fish species, species of the *Sebastidae* family are characterized by the presence of sharp spines on their dorsal fins. We suspected that sensitization through injuries caused by sharp fins may be one cause of allergies specific to the *Sebastidae* family. However, our patient had no history of frequent contact with fish or injury from fish spines. The route of sensitization to *Sebastidae* in this case could not be clearly identified. Although the patient and their guardian reported no prior history of consuming *Sebastidae*, the possibility of unrecognized oral exposure cannot be completely excluded, as mislabeling or cross-contamination of fish products may occur in daily dietary settings.

Several proteins have been identified as the causative allergens of fish allergy, including parvalbumin, enolase, aldolase, tropomyosin, myoglobin, collagen, and creatine kinase [1, 3]. Parvalbumin, the most well-recognized fish allergens, is a calcium-binding protein of approximately 12 kDa that is widely distributed in bony fishes and plays a role in muscle contraction. Different fish species express distinct isoforms of parvalbumin, such as Gad c 1 in cod and Seb m 1 in *Sebastes* species [5]. In fish allergy to *Sebastes* species, Seb m 1 may contribute to sensitization. Although parvalbumin exhibits slight differences in amino acid sequences among fish species, it retains high structural similarity and exhibits strong cross-reactivity between fish species [1]. Therefore, various fish species can cause allergic reactions in patients sensitized to parvalbumin. Although differences in parvalbumin content among fish species and tissue types can modify the severity of allergic symptoms [6, 7], the presence of patients with food allergy who react to only a limited number of fish species suggests the involvement of species-specific allergens other than parvalbumin [8, 9]. In this case, the band detected by immunoblot assay was not 12 kDa, suggesting that parvalbumin was not the causative allergen. In addition, a specific IgE test in the patient’s serum against Gad c 1, the parvalbumin of cod (*Gadus callarias*), was negative.

In this case, proteins with molecular weights of approximately 17 and 110 kDa that reacted exclusively with species of the *Sebastidae* family were identified, and five proteins were identified as potential allergen candidates by mass spectrometry. Protein identification was based on predicted sequences and homology to known proteins, and therefore species specificity should be interpreted with caution. ATPase sarcoplasmic/endoplasmic reticulum Ca2 + transporting1-like isoform X1 (SERCA1), actively transports calcium ions to the sarcoplasmic reticulum and plays a crucial role in regulating muscle contraction and relaxation. While there are no prior reports of SERCA1 as a fish allergen, it has been identified as an allergen in crustaceans, such as *Chionoecetes opilio* (snow crab) [10]. Similarly, alpha-actinin-3b is a key structural component of muscle fibers that binds to and stabilizes α-actin. Notably, alpha-actin has been reported as a fish allergen [11]. Although reports of alpha-actinin-3b as a food allergen are limited, it has been implicated in occupational allergies related to fish processing, where repeated exposure to fish leads to sensitization [12]. Myosin light chain, phosphorylatable, fast skeletal muscle a (myosin light chain 2) and fast skeletal muscle myosin light polypeptide 3 (myosin light chain 3) are essential proteins controlling muscle contraction mechanisms and structural stability and regulate contraction speed and intensity. Cases of myosin light chain sensitization in cod-allergic patients have been reported [13], and other studies have reported that 71% of patients with fish allergy exhibited sensitization to myosin light chains [14]. Given that sensitization to myosin light chains has been reported in a substantial proportion of patients with fish allergy, it is unlikely that these proteins represent a species-specific allergen for *Sebastidae* family. In addition to its identification in fish, myosin light chain has also been reported as an IgE-binding protein in crustacean shellfish allergy, indicating that it is not unique to fish species [15]. Nucleoside diphosphate kinase B-like catalyzes phosphate group transfer between nucleotides and plays a role in energy metabolism, cell signaling, and the immune response; however, there have been no reports of nucleoside diphosphate kinase B-like as a fish allergen. Taken together, although definitive identification of the causative allergen was not possible, α-actinin-3b and SERCA1 may represent relatively more likely candidates in this case. Further studies, including recombinant protein–based IgE-binding assays and functional assays such as basophil activation tests, are required to confirm their roles as IgE-reactive and functionally active allergens.

One possible reason for allergic reactions occurring specifically with fish of the *Sebastidae* family is that they may contain higher concentrations of the causative proteins compared with other fish species. All four proteins, except nucleoside diphosphate kinase B-like, are known to be abundant in fast-twitch muscle fibers, and fishes of the *Sebastidae* family have higher fast-twitch muscle content than other fish species. In this case report, immunoblot analysis with specific antibodies against each protein could not be performed. Future studies are therefore required to compare the protein levels among various fish species using specific antibodies against these proteins. Another potential reason could be the differences in amino acid sequence homology, post-translational modifications, or three-dimensional structure of the causative proteins, which could enhance the allergenicity of fish in the *Sebastidae* family.

This case report has several limitations. First, we were unable to perform an oral food challenge, which is the gold standard for confirming both the diagnosis of allergy to fish belonging to the *Sebastidae* family and tolerance to other fish. Second, a 6-month follow-up period after eliminating fish belonging to the *Sebastidae* family from the diet may be insufficient to assess the risk of recurrence, and longer-term observation is necessary. Third, only raw fish extracts were tested in the skin prick test, BAT, and immunoblot analyses; therefore, we could not assess whether heat-induced structural changes may have influenced IgE binding. Fourth, the BAT was conducted only at relatively high antigen concentrations, and a reliable dose–response curve could not be tested. Fifth, the lack of specific IgE antibody testing against parvalbumin of the *Sebastidae* family did not directly prove non-sensitization to parvalbumin. Finally, although a 1:10 serum dilution for IgE immunoblotting was selected with reference to previous allergen characterization studies, further optimization through serial dilution would have been preferable. The candidate proteins identified in this case report are based on a single case, and further investigations involving additional cases are necessary to confirm their roles as allergens.

In conclusion, we report a pediatric case of fish allergy in which the patient reacted exclusively to fish species from the *Sebastidae* family. We detected several proteins other than parvalbumin with serum IgE reactivity. This case demonstrates the need for clinicians to recognize fish allergy specific to the *Sebastidae* family. Identifying the causative allergens will improve understanding of fish allergy diversity and individualized dietary management for patient. Additional cases and detailed analyses are necessary to identify the specific allergens involved in *Sebastidae* allergy.

## Data Availability

All data generated during this study are included in this published article. Additional details may be available from the corresponding author on reasonable request considering patient privacy.

## Abbreviations

BAT: Basophil Activation Test
IgE: Immunoglobulin E
SPT: Skin prick test
